# Hyperthyroidism as a reversible cause of right ventricular overload and congestive heart failure

**DOI:** 10.1186/1476-7120-6-29

**Published:** 2008-06-12

**Authors:** Raniero Di Giovambattista

**Affiliations:** 1 Cardiology Division, Department of Medicine, Hospital of Avezzano, Italy

## Abstract

We describe a case of severe congestive heart failure and right ventricular overload associated with overt hyperthyroidism, completely reversed with antithyroid therapy in a few week. It represents a very unusual presentation of overt hyperthyroidism because of the severity of right heart failure. The impressive right ventricular volume overload made mandatory to perform transesophageal echo and angio-TC examination to exclude the coexistence of ASD or anomalous pulmonary venous return. Only a few cases of reversible right heart failure, with or without pulmonary hypertension, have been reported worldwide. In our case the most striking feature has been the normalization of the cardiovascular findings after six weeks of tiamazole therapy.

## Background

Congestive heart failure (CHF) represents the initial clinical presentation in approximately 6% of patients with overt hyperthyroidism, and half of them have LV dysfunction [1]. Symptoms of CHF most often subside and LVEF improves following treatment of hyperthyroidism [2]. Nonetheless one-third of these patients develops persisted dilated cardiomyopathy [1]. Overt hyperthyroidism as a cause of reversible pulmonary hypertension is also reported in the literature [3]. This finding might be explained, at least in part, by the effect of thyroid hormone on lowering peripheral resistance and increasing cardiac output and pulmonary flow. Few case reports however describe isolated right heart failure, severe right ventricle volume overload and tricuspid regurgitation as a consequence of a hyperthyroidism [4-9]. Most textbooks do not mention at all hyperthyroidism as a cause of isolated right ventricular failure. We report the case of a 51 year old woman affected by overt hyperthyroidism presenting with severe right heart failure and echocardiographic finding of right ventricle volume overload (ie paradoxical septal motion, severe tricuspid regurgitation, RV dilatation) which have been promptly resolved with hyperthyroidism treatment.

## Case presentation

N.T., a 54-year-old woman born in Ukraine and resident up to 2001 in a small village 400 km from Chernobyl, was admitted to our hospital in Avezzano, Italy in February 2005 for unexplained, progressive, severe dyspnea. In September 2003, she had the diagnosis of clinical hyperthyroidism, possibly due to radiation fall-out after the Chernobyl explosion (in 1986), and mild essential hypertension; she was in stable hemodynamic and cardiac conditions and discharged with beta-blockers, ramipril, and tiamazole therapy.

**Figure 1:**
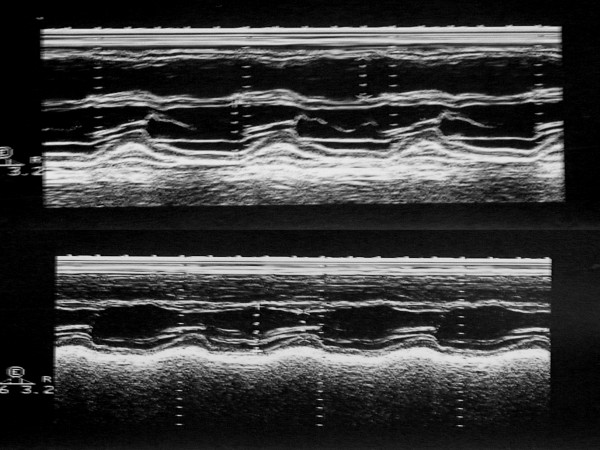
2-D targeted M-mode tracings of the left ventricle upon admission (with overt hyperthyroidism) and after 6 weeks (with euthyroid state following thyreostatic therapy). The dilated, hyperkinetic right heart on admission, with paradoxical septal movement and mild pericardial effusion, becomes completely normal after 6 weeks.

On February 2005, she spontaneously withdrew from medical therapy. At that time, she was working, with an irregular clandestine administrative position, in an Italian family as a "badante", taking care of an elderly and had no regular immigration visa. This condition restricted her from seeking medical advice. In February 2005, after spontaneous medical therapy withdrawal, she developed progressive asthenia, dyspnea, weight loss (10 kilograms in the last 2 months), and tremors. She came to the emergency room with obligatory semi-orthopnoic decubitus, anasarcatic condition with severe edema, ascites and bilateral pleural effusion with small pericardial effusion. EKG showed atrial fibrillation with rapid ventricular response (110 beats per minute). Transthoracic echocardiography showed a dilated, hyperkinetic right ventricle, with tricuspid anulus posterior septal excursion of 28 mm, a well contracting left ventricle, paradoxical movement of interventricular septum and severe tricuspid insufficiency without pulmonary hypertension. (Fig 1). Laboratory findings are summarized in Table 1 and appear to be consistent with severe hyperthyroidism. The patient started with tiamazole 15 mg a day, diuretics (furosemide), beta-blockers and low molecular weight heparin; sinus rhythm was restored after 48 hours. Right ventricular overload persisted also in sinus rhythm. We decided to perform angio-computerized tomography and TEE contrast echo, both negative, to rule out an interatrial defect or anomalous venous return potentially responsible of the right ventricular volume overload. There was a gradual improvement of all clinical symptoms and laboratory findings with full normalization of the echocardiographic findings after 6 weeks of therapy (Fig 1). Antithyroid therapy was finally withdrown without any recurrence of hyperthyroidism at 3 and 6 months follow-up.

**Table 1:** Results of Hematologic and Serum Chemical Laboratory Tests

Test	March 17th,	March 25th	April 6th	May 25th
TSH	< 0.01	< 0.01	< 0.01	0.92
FT3	32.53	4.33		3.85
FT4	12.75	2.38		0.95
AbTG	78			
Ab PRS	293			
BNP	1,420	1,577	766	249
Creatinine	0.2	0.3	0.2	0.6
Albumin	2.6		2.3	4.1
Lactic Acid	2.8			
Pseudocholinesterasis	1,819			
Total Cholesterol	76	98	96	200
Total Bilirubin	7.8	9.53	7.9	
ALT	48			38
AST	54			38
White-cell count	4,400			5,150
ESR	8			
PCR	3			

Reference range: TSH 0.35–4.94 (mU/ml), FT3 1.71–3.71 pg/ml, FT4 0.70–1.48 ng/dl, AbTG – 0–28.7 mU/ml, BNP < 100 pg/ml, Creatinine 0.7–1.2 mg/ml, Albumin 3.5–5.0 gr/dl, Lactic Acid-0.7–2.1 mmol/l, Pseudocholinesterasis 4,650–12,220 U/l, Total Cholesterol 100–200 mg/dl, Total Bilirubin 0.2–1.3 mg/dl, ALT 7–56 U/l, AST 15–46 U/l, White-cell count 4,600–10,200 mm^3^, ESR – 1–15 mm/h, PCR < 5 mg/l

## Discussion

Palpitation, dyspnea and cough are the more common symptoms among patients with overt hyperthyroidism in a matched case-control study involving a total of 393 patients in England [10]. Palpitation generally correlates with new onset atrial fibrillation or atrial tachycardia matched with increased heart contractility. In the presence of CAD, angina may develop as a consequence of imbalance between myocardial O_2 _demand and supply. The increased cardiac contractile function of patients with hyperthyroidism makes the development of heart failure unexpected and raises the question of hyperthyroid cardiomyopathy. Patients may occasionally have exertional dyspnea due, at least in part, to the subnormal response to exercise as a result of the inability to increase heart rate maximally or to lower vascular resistances further during, as normally occurs, with exercise. In many cases the overt hyperthyroid state is characterized by a high LVEF at rest with a paradoxical, but significant fall in LVEF during exercise. [11] Hyperthyroid cardiovascular system is highly stressed at rest and its functional reserve is reduced. [12,13]. Congestive heart failure however is very rare and, when present, is generally associated with atrial fibrillation, more advanced age, persistent systolic LV dysfunction. [1] Our case report describes a very unusual presentation of overt hyperthyroidism because of the presence and the severity of right heart failure with an impressive right ventricle volume overload which made mandatory to perform transoesophageal echo and angio CT examination to exclude the coexistence of ASD or anomalous pulmonary venous return. No signs of LV dysfunction were present. Only a few cases of reversible right heart failure, with or without pulmonary hypertension, have been reported worldwide. Saad et al. [5] recently described a similar case regarding a young women with Graves-Basedow disease, without history of cardiovascular disease, who complained about palpitation, peripheral edema, weight loss and fever. The chest x-ray and the echocardiogram showed right ventricular dilatation and severe tricuspid regurgitation without pulmonary hypertension. Right ventricular dysfunction disappeared after therapy with propanolol, corticosteroids and diuretics. In our case the most striking feature has been the normalization of the cardiovascular findings after six weeks of tiamazole therapy. The exact reasons for the development of right ventricle volume overload in hyperthyroidism are yet unclear. Cardiovascular manifestations of hyperthyroidism are frequent and sometimes are relevant in the clinical picture. Usually a hyper-dynamic circulatory state hallmarks the disease with low peripheral resistance, increased output, possibly with pulmonary hypertension as a consequence of increased pulmonary flow. A study published by Merce J et al. in 2005 found a high prevalence of pulmonary hypertension in hyperthyroidism, which was corrected after treatment [3]. However we found a more unusual presentation with normal pulmonary pressure, right chamber dilatation, massive tricuspid regurgitation, ascites and bilateral pleural effusion. Mechanisms more often invoked and at least in part above described, such as increased cardiac output and venous return, high cardiac output-induced endothelial pulmonary injury, may have act in our patient for a time long enough to provoke right ventricle dilatation and functional tricuspid regurgitation in a well known vicious cycle towards right heart failure.

## Conclusion

We describe a case of right ventricular overload associated with overt hyperthyroidism, completely reversed with antithyroid therapy in a few weeks. Our case report can be of interest for the cardiologist and the echocardiographer. For the cardiologist, this case is a reminder that there is a reversible, curable, and – if recognized – benign cause of congestive heart failure and right ventricular overload. Therefore, thyroid function should always be considered as a cause of cardiac dysfunction, especially when the clinical picture is consistent with a high output state. For the sonographer, this case can also be teaching, since there was an echocardiographic syndrome of right ventricular volume overload with paradoxical septal movement and normal-increased TAPSE. The usual causes considered in the differential diagnosis are primitive or secondary (tricuspid and pulmonary) valvular insufficiency, or left-to right shunting due to interatrial defect or anomalous venous return. The identification of a sinus venous type of interatrial defect and partial anomalous venous return can be especially challenging with transthoracic echocardiography, and we had to perform a contrast TEE and an angio-CT to be on the safe side in excluding these potentially curable conditions which may have a clinical onset in the adult or even more advanced age. However, this case reminds us that there is an additional condition for a dominant right ventricular volume overload and right heart failure in the absence of any detectable shunt or valvular alterations. The right heart can be the victim of thyroid dysfunction, with a picture of right ventricular overload which may dominate over the more frequently described left ventricular failure due to left heart high output failure [1,2,10-12].

## Consent

Written informed consent was obtained from the patient's next-of-kin for publication of this case report and accompanying images. A copy of the written consent is available for review by the Editor-in-Chief of this journal.
